# Reply to Comments by Yih et al. (Exposure to Hantavirus is a Risk Factor Associated with Kidney Diseases in Sri Lanka: A Cross-Sectional Study)

**DOI:** 10.3390/v11121150

**Published:** 2019-12-11

**Authors:** Yomani D. Sarathkumara, Chandika D. Gamage, Sithumini Lokupathirage, Devinda S. Muthusinghe, Nishantha Nanayakkara, Lishanthe Gunarathne, Kenta Shimizu, Yoshimi Tsuda, Jiro Arikawa, Kumiko Yoshimatsu

**Affiliations:** 1 Department of Microbiology, Faculty of Medicine, University of Peradeniya, Peradeniya 20400, Sri Lanka; yomani_ds@yahoo.com (Y.D.S.); chandika@pdn.ac.lk (C.D.G.); 2 Department of Microbiology and Immunology, Faculty of Medicine, Hokkaido University, Sapporo 060-8638, Japan; sithuminim@med.hokudai.ac.jp (S.L.); kshimizu@med.hokudai.ac.jp (K.S.); tsuday@med.hokudai.ac.jp (Y.T.); j_arika@med.hokudai.ac.jp (J.A.); 3 Graduate School of Infectious Diseases, Hokkaido University, Sapporo 060-8638, Japan; shameera.dev@gmail.com; 4 Nephrology and Transplantation Unit, Teaching Hospital Kandy, Kandy 20000, Sri Lanka; nishantha4313@gmail.com; 5 Renal Clinic, District Hospital, Girandurukotte 90750, Sri Lanka; lishanthe@yahoo.co.uk

Dear Drs. W. Katherine Yih, Martin Kulldorff, Jessica H. Leibler, David J. Friedman, and Daniel R. Brooks,

Thank you for your interest in our research. Please find supplementary data with the results of multivariate logistic regression analyses. Drs. Yomani D Sarathukumara and Chandika D Gamage reanalyzed the data according to your suggestions. If you have additional questions, please feel free to contact us.

In 2017, we reported high seroprevelance of 54.5% for hantavirus antibodies among CKDu patients than healthy controls (13.5%) from a CKDu hotspot, Girandurukotte [1]. It was the first evidence of exposure to hanatavirus infection in CKDu patients in Sri Lanka. Prior conducting the current study, we performed a preliminary study using archived sera samples and epidemiological data collected by Nanayakkara et al [2]. They had a large collection of data on information about occupation, smoking, drinking, chewing betel nuts, various medical parameters, and so forth incuding blood samples from patients and healthy controls from two CKDu hotspots of Sri Lanka namely Girandurukotte and Medawachchiya. In collaboration with a research group at Kyoto University, we tested aforementioned sera samples (approximately 600) for anti-hantarvirus antibodies. The antibody-positive rate was almost similar as previously reported [1]. The first author of this study, Dr. S. Nanayakkara, statistically analyzed the data and found no significancant differeneces between the epidemiological data collected and anti-hantavirus antibody-positivity, although a significant association was found being a CKDu patient. On the other hand, their epdidemiological data had no information related to rodent-borne infectious diseases when considering their association with hantavirus infection. Samples were collected only from men from the two CKDu hostpots. To overcome these limitations and based on these preliminary results, we implemented the current study solely aimed to investigate the “exposure to hanativirus infection among CKDu patients and controls and the factors that are associated between hantavirus infection and CKDu”.

Furthermore, we were interested in comparing the exposure to hanatiavirus in a CKDu enedemic vs non-endemic area in the country since CKDu is highly prevelaent in the rice farming communities in the dry climatic zones of Sri Lanka. In addition, dry climatic factors influence hanativirus disease transmission dynamics. Therefore, we selected Girandurukotte as a CKDu hostpot in the dry climatic zone whilst Kandy was selected as non-CKDu endemic area belonging to the wet climatic zone of Sri Lanka.

Age, gender and occupation are known risk factors for CKDu in Sri Lanka. Many global epidemiological studies have also identified similar risk factos for hantavirus infection. Hence, risk factors of CKDu and hantavirus infections are considered to be overlapped. Based on the pathological studies on renal morphology shows similar characteristics on CKDu and in renal syndromes caused by hantaviruses. Despite the statistical interpretation of sero-epidemiological results, the unusual elevated seroprevalence reported among CKDu patients and healthy controls is very concerning. In Vietnam, the antibody-positive rate is around 5% in infected harbor workers. It is about 0.8% even for unknown fever patients in Thailand. However, we believe that it is necessary to clarify the cause of high seroprevalence of hantavirus in Sri Lanka.

However, your thought-provoking, informative article [3] is of great help to us. Tropical Mesoamerican nephropathy found in Central American countries is a concerning public health problem with a global importance. We have experienced and witnessed the tragic situation of CKDu in Sri Lanka in a similar manner during field visits to collect blood samples and to capture the source of infection. Most of the CKDu patients are from the very remote areas of Sri Lanka. They lack dialysis facilities in the hospital as they require to travel more than 100 km to the nearest hospital that facilitates dialysis and renal biopsies or other advanced medical treatments. Most of the individuals living in CKDu hostpots are paddy farmers. These farmers are usually exposed to rats and mice.

As future aspects of this study, we are planning to investigate the hantavirus sero-prevelance and infective setotypes in an island-wide epidemiological study. We also have results showing that there is no association between CKDu and leptospirosis in Sri Lanka. However, the sero-diagnosis of leptospirosis is problematic when performing MAT as the gold standard method of diagnosis, as you know, and has not yet published.

If hantavirus infection is a potential risk of CKDu in Sri Lanka and Central Americans countries, novel prevention methods should be considered. However, these unexpected seroprevelances of hantaviruses is a prevailing consideration to improve the tragic situation in Sri Lanka. We would like to strongly recommend you that to use other hantavirus antigens for serological surveys, which should be nonpathogenic, and Central American strains such as El Moro Canyon, Cano delgadito, and others. If you need any assistance, Dr. Yoshimatsu will be able to help you.

## Supplementary Materials

Supplementary File 1
