# Supplementary material for: Reply to Comments by Yih et al. (Exposure to Hantavirus is a Risk Factor Associated with Kidney Diseases in Sri Lanka: A Cross-Sectional Study)
Source: Viruses. 2019 Dec 11;11(12):1150. doi: 10.3390/v11121150 (PMC6949920; doi:10.3390/v11121150)
Supplement: Supplementary File 1 [file viruses-11-01150-s001.zip › supplementary data/CKDu Binarylogistic-Kandy-All.pdf]

```
LOGISTIC REGRESSION VARIABLES Renal_patient
  /METHOD=ENTER Q2_Age
  /METHOD=ENTER Male
  /METHOD=ENTER Farming1_0
  /METHOD=ENTER RatsAtHome1_0
  /METHOD=ENTER DriedRodentFaeces1_0
  /METHOD=ENTER Hanta_positive
  /CONTRAST (Male)=Indicator(1)
  /CONTRAST (Farming1_0)=Indicator(1)
  /CONTRAST (RatsAtHome1_0)=Indicator(1)
  /CONTRAST (DriedRodentFaeces1_0)=Indicator(1)
  /CONTRAST (Hanta_positive)=Indicator(1)
  /PRINT=GOODFIT CI(95)
  /CRITERIA=PIN(0.05) POUT(0.10) ITERATE(20) CUT(0.5).
```

Logistic Regression

[DataSet2] C:\Users\Chandika\Documents\yomani-MPhil thesis\Binarary Logistics \Kandy\Kandy_All.sav

Case Processing Summary

Unweighted Cases[a]		N	Percent
Selected Cases	Included in Analysis	320	100.0
	Missing Cases	0	.0
	Total	320	100.0
Unselected Cases		0	.0
Total		320	100.0

a. If weight is in effect, see classification table for the total number of cases.

Dependent Variable Encoding

Original Value	Internal Value
0	0
1	1

Categorical Variables Codings

		Frequency	Parameter coding (1)
Hanta_positive?	0	292	.000
	1	28	1.000
Farming1_0	0	251	.000
	1	69	1.000
RatsAtHome1_0	0	81	.000
	1	239	1.000
DriedRodentFaeces1_0	0	195	.000
	1	125	1.000
Male?	0	166	.000
	1	154	1.000

Block 0: Beginning Block

Classification Table[a,b]

	Observed		Predicted Renal_patient? 0	Predicted Renal_patient? 1	Predicted Percentage Correct
Step 0	Renal_patient?	0	270	0	100.0
		1	50	0	.0
	Overall Percentage				84.4

a. Constant is included in the model.

b. The cut value is .500

Variables in the Equation

		B	S.E.	Wald	df	Sig.	Exp(B)
Step 0	Constant	-1.686	.154	119.979	1	.000	.185

Variables not in the Equation

			Score	df	Sig.
Step 0	Variables	Q2_Age	10.610	1	.001
	Overall Statistics		10.610	1	.001

Block 1: Method = Enter

Omnibus Tests of Model Coefficients

		Chi-square	df	Sig.
Step 1	Step	10.585	1	.001
	Block	10.585	1	.001
	Model	10.585	1	.001

Model Summary

Step	-2 Log likelihood	Cox & Snell R Square	Nagelkerke R Square
1	266.791[a]	.033	.056

a. Estimation terminated at iteration number 5 because parameter estimates changed by less than .001.

Hosmer and Lemeshow Test

Step	Chi-square	df	Sig.
1	10.029	8	.263

Contingency Table for Hosmer and Lemeshow Test

		Renal_patient? = 0		Renal_patient? = 1		
		Observed	Expected	Observed	Expected	Total
Step 1	1	34	31.619	0	2.381	34
	2	32	30.838	2	3.162	34
	3	31	32.989	6	4.011	37
	4	22	24.575	6	3.425	28
	5	24	25.054	5	3.946	29
	6	27	26.385	4	4.615	31
	7	27	27.396	6	5.604	33
	8	28	25.664	4	6.336	32
	9	21	23.750	10	7.250	31
	10	24	21.729	7	9.271	31

Classification Table[a]

			Predicted		
			Renal_patient?		Percentage Correct
Observed			0	1	
Step 1	Renal_patient?	0	270	0	100.0
		1	50	0	.0
	Overall Percentage				84.4

a. The cut value is .500

Variables in the Equation

		B	S.E.	Wald	df	Sig.	Exp(B)	95% C.I....
								Lower
Step 1[a]	Q2_Age	.036	.011	10.194	1	.001	1.037	1.014
	Constant	-3.520	.620	32.218	1	.000	.030	

Variables in the Equation

		95% C.I....
		Upper
Step 1[a]	Q2_Age	1.061
	Constant	

a. Variable(s) entered on step 1: Q2_Age.

Block 2: Method = Enter

Omnibus Tests of Model Coefficients

		Chi-square	df	Sig.
Step 1	Step	1.066	1	.302
	Block	1.066	1	.302
	Model	11.651	2	.003

Model Summary

Step	-2 Log likelihood	Cox & Snell R Square	Nagelkerke R Square
1	265.724[a]	.036	.062

a. Estimation terminated at iteration number 5 because parameter estimates changed by less than .001.

Hosmer and Lemeshow Test

Step	Chi-square	df	Sig.
1	10.720	8	.218

Contingency Table for Hosmer and Lemeshow Test

		Renal_patient? = 0		Renal_patient? = 1		Total
		Observed	Expected	Observed	Expected	
Step 1	1	33	30.777	0	2.223	33
	2	29	27.369	1	2.631	30
	3	29	29.574	4	3.426	33
	4	28	30.789	7	4.211	35
	5	26	27.777	6	4.223	32
	6	24	26.356	7	4.644	31
	7	30	27.403	3	5.597	33
	8	23	24.009	7	5.991	30
	9	27	24.533	5	7.467	32
	10	21	21.413	10	9.587	31

Classification Table[a]

Observed			Predicted		
			Renal_patient?		Percentage Correct
			0	1	
Step 1	Renal_patient?	0	270	0	100.0
		1	50	0	.0
	Overall Percentage				84.4

a. The cut value is .500

Variables in the Equation

		B	S.E.	Wald	df	Sig.	Exp(B)	95% C.I....
								Lower
Step 1[a]	Q2_Age	.036	.011	9.800	1	.002	1.036	1.013
	Male(1)	.325	.315	1.061	1	.303	1.384	.746
	Constant	-3.651	.635	33.011	1	.000	.026	

Variables in the Equation

		95% C.I....
		Upper
Step 1[a]	Q2_Age	1.060
	Male(1)	2.568
	Constant	

a. Variable(s) entered on step 1: Male.

Block 3: Method = Enter

Omnibus Tests of Model Coefficients

		Chi-square	df	Sig.
Step 1	Step	.008	1	.929
	Block	.008	1	.929
	Model	11.659	3	.009

Model Summary

Step	-2 Log likelihood	Cox & Snell R Square	Nagelkerke R Square
1	265.716[a]	.036	.062

a. Estimation terminated at iteration number 5 because parameter estimates changed by less than .001.

Hosmer and Lemeshow Test

Step	Chi-square	df	Sig.
1	8.994	8	.343

Contingency Table for Hosmer and Lemeshow Test

		Renal_patient? = 0		Renal_patient? = 1		
		Observed	Expected	Observed	Expected	Total
Step 1	1	32	29.859	0	2.141	32
	2	31	29.192	1	2.808	32
	3	28	28.671	4	3.329	32
	4	28	30.794	7	4.206	35
	5	26	27.772	6	4.228	32
	6	25	27.206	7	4.794	32
	7	29	27.390	4	5.610	33
	8	28	27.082	6	6.918	34
	9	25	23.565	6	7.435	31
	10	18	18.468	9	8.532	27

Classification Table[a]

			Predicted		
			Renal_patient?		Percentage Correct
	Observed		0	1	
Step 1	Renal_patient?	0	270	0	100.0
		1	50	0	.0
	Overall Percentage				84.4

a. The cut value is .500

Variables in the Equation

		B	S.E.	Wald	df	Sig.	Exp(B)
Step 1[a]	Q2_Age	.036	.012	9.457	1	.002	1.037
	Male(1)	.330	.321	1.059	1	.303	1.391
	Farming1_0(1)	-.034	.379	.008	1	.929	.967
	Constant	-3.657	.640	32.702	1	.000	.026

Variables in the Equation

		95% C.I.for EXP(B)	
		Lower	Upper
Step 1[a]	Q2_Age	1.013	1.061
	Male(1)	.742	2.609
	Farming1_0(1)	.460	2.032
	Constant		

a. Variable(s) entered on step 1: Farming1_0.

Block 4: Method = Enter

Omnibus Tests of Model Coefficients

		Chi-square	df	Sig.
Step 1	Step	2.600	1	.107
	Block	2.600	1	.107
	Model	14.259	4	.007

Model Summary

Step	-2 Log likelihood	Cox & Snell R Square	Nagelkerke R Square
1	263.116[a]	.044	.075

a. Estimation terminated at iteration number 5 because parameter estimates changed by less than .001.

Hosmer and Lemeshow Test

Step	Chi-square	df	Sig.
1	8.862	8	.354

Contingency Table for Hosmer and Lemeshow Test

		Renal_patient? = 0		Renal_patient? = 1		
		Observed	Expected	Observed	Expected	Total
Step 1	1	32	30.009	0	1.991	32
	2	31	29.313	1	2.687	32
	3	29	28.811	3	3.189	32
	4	28	28.380	4	3.620	32
	5	25	27.922	7	4.078	32
	6	26	27.339	6	4.661	32
	7	25	27.342	8	5.658	33
	8	27	26.437	6	6.563	33
	9	24	24.507	8	7.493	32
	10	23	19.939	7	10.061	30

Classification Table[a]

			Predicted		
			Renal_patient?		Percentage Correct
Observed			0	1	
Step 1	Renal_patient?	0	270	0	100.0
		1	50	0	.0
	Overall Percentage				84.4

a. The cut value is .500

Variables in the Equation

		B	S.E.	Wald	df	Sig.	Exp(B)
Step 1[a]	Q2_Age	.035	.012	9.077	1	.003	1.036
	Male(1)	.386	.326	1.407	1	.236	1.471
	Farming1_0(1)	.016	.385	.002	1	.968	1.016
	RatsAtHome1_0(1)	-.565	.344	2.693	1	.101	.568
	Constant	-3.255	.674	23.319	1	.000	.039

Variables in the Equation

		95% C.I.for EXP(B)	
		Lower	Upper
Step 1[a]	Q2_Age	1.012	1.060
	Male(1)	.777	2.785
	Farming1_0(1)	.477	2.162
	RatsAtHome1_0(1)	.289	1.116
	Constant		

a. Variable(s) entered on step 1: RatsAtHome1_0.

Block 5: Method = Enter

Omnibus Tests of Model Coefficients

		Chi-square	df	Sig.
Step 1	Step	.000	1	.986
	Block	.000	1	.986
	Model	14.260	5	.014

Model Summary

Step	-2 Log likelihood	Cox & Snell R Square	Nagelkerke R Square
1	263.116[a]	.044	.075

a. Estimation terminated at iteration number 5 because parameter estimates changed by less than .001.

Hosmer and Lemeshow Test

Step	Chi-square	df	Sig.
1	9.875	8	.274

Contingency Table for Hosmer and Lemeshow Test

		Renal_patient? = 0		Renal_patient? = 1		
		Observed	Expected	Observed	Expected	Total
Step 1	1	32	30.010	0	1.990	32
	2	31	29.313	1	2.687	32
	3	29	28.811	3	3.189	32
	4	28	28.380	4	3.620	32
	5	25	28.784	8	4.216	33
	6	27	27.317	5	4.683	32
	7	25	27.317	8	5.683	33
	8	26	25.622	6	6.378	32
	9	24	24.512	8	7.488	32
	10	23	19.935	7	10.065	30

Classification Table[a]

			Predicted		
			Renal_patient?		Percentage Correct
	Observed		0	1	
Step 1	Renal_patient?	0	270	0	100.0
		1	50	0	.0
	Overall Percentage				84.4

a. The cut value is .500

Variables in the Equation

		B	S.E.	Wald	df	Sig.	Exp(B)
Step 1[a]	Q2_Age	.035	.012	9.005	1	.003	1.036
	Male(1)	.387	.326	1.403	1	.236	1.472
	Farming1_0(1)	.015	.388	.001	1	.970	1.015
	RatsAtHome1_0(1)	-.568	.376	2.275	1	.131	.567
	DriedRodentFaeces1_0(1)	.007	.366	.000	1	.986	1.007
	Constant	-3.257	.679	22.983	1	.000	.039

Variables in the Equation

		95% C.I.for EXP(B)	
		Lower	Upper
Step 1[a]	Q2_Age	1.012	1.060
	Male(1)	.776	2.790
	Farming1_0(1)	.474	2.173
	RatsAtHome1_0(1)	.271	1.185
	DriedRodentFaeces1_0(1)	.492	2.061
	Constant		

a. Variable(s) entered on step 1: DriedRodentFaeces1_0.

Block 6: Method = Enter

Omnibus Tests of Model Coefficients

		Chi-square	df	Sig.
Step 1	Step	3.170	1	.075
	Block	3.170	1	.075
	Model	17.429	6	.008

Model Summary

Step	-2 Log likelihood	Cox & Snell R Square	Nagelkerke R Square
1	259.946[a]	.053	.091

a. Estimation terminated at iteration number 5 because parameter estimates changed by less than .001.

Hosmer and Lemeshow Test

Step	Chi-square	df	Sig.
1	5.733	8	.677

Contingency Table for Hosmer and Lemeshow Test

		Renal_patient? = 0		Renal_patient? = 1		
		Observed	Expected	Observed	Expected	Total
Step 1	1	32	30.032	0	1.968	32
	2	32	30.283	1	2.717	33
	3	30	29.816	3	3.184	33
	4	27	28.510	5	3.490	32
	5	27	28.032	5	3.968	32
	6	26	27.452	6	4.548	32
	7	28	27.448	5	5.552	33
	8	24	25.785	8	6.215	32
	9	25	24.566	7	7.434	32
	10	19	18.076	10	10.924	29

Classification Table[a]

			Predicted		
			Renal_patient?		Percentage Correct
Observed			0	1	
Step 1	Renal_patient?	0	268	2	99.3
		1	49	1	2.0
	Overall Percentage				84.1

a. The cut value is .500

Variables in the Equation

		B	S.E.	Wald	df	Sig.	Exp(B)
Step 1[a]	Q2_Age	.034	.012	8.077	1	.004	1.034
	Male(1)	.341	.329	1.078	1	.299	1.407
	Farming1_0(1)	-.100	.400	.062	1	.803	.905
	RatsAtHome1_0(1)	-.538	.381	2.001	1	.157	.584
	DriedRodentFaeces1_0(1)	.004	.368	.000	1	.992	1.004
	Hanta_positive(1)	.868	.469	3.427	1	.064	2.381
	Constant	-3.241	.679	22.803	1	.000	.039

Variables in the Equation

		95% C.I.for EXP(B)	
		Lower	Upper
Step 1[a]	Q2_Age	1.010	1.058
	Male(1)	.738	2.681
	Farming1_0(1)	.413	1.982
	RatsAtHome1_0(1)	.277	1.231
	DriedRodentFaeces1_0(1)	.488	2.064
	Hanta_positive(1)	.950	5.968
	Constant		

a. Variable(s) entered on step 1: Hanta_positive.
