# Supplementary material for: Reply to Comments by Yih et al. (Exposure to Hantavirus is a Risk Factor Associated with Kidney Diseases in Sri Lanka: A Cross-Sectional Study)
Source: Viruses. 2019 Dec 11;11(12):1150. doi: 10.3390/v11121150 (PMC6949920; doi:10.3390/v11121150)
Supplement: Supplementary File 1 [file viruses-11-01150-s001.zip › supplementary data/CKDuBinaryLogistic-GK_all.pdf]

```
LOGISTIC REGRESSION VARIABLES Renal_patient
  /METHOD=ENTER Q2_Age
  /METHOD=ENTER Q3_Gender
  /METHOD=ENTER Farming
  /METHOD=ENTER RatsInHouse
  /METHOD=ENTER DriedRodentFaeces2
  /METHOD=ENTER Hanta_positive
  /CONTRAST (Q3_Gender)=Indicator(1)
  /CONTRAST (Farming)=Indicator(1)
  /CONTRAST (RatsInHouse)=Indicator(1)
  /CONTRAST (DriedRodentFaeces2)=Indicator(1)
  /PRINT=GOODFIT CI(95)
  /CRITERIA=PIN(0.05) POUT(0.10) ITERATE(20) CUT(0.5).
```

Logistic Regression

[DataSet1] C:\Users\Chandika\Documents\yomani-MPhil thesis\Binarary Logistics\GK-all-cases.sav

Case Processing Summary

Unweighted Cases[a]		N	Percent
Selected Cases	Included in Analysis	346	100.0
	Missing Cases	0	.0
	Total	346	100.0
Unselected Cases		0	.0
Total		346	100.0

a. If weight is in effect, see classification table for the total number of cases.

Dependent Variable Encoding

Original Value	Internal Value
0	0
1	1

Categorical Variables Codings

		Frequency	Parameter coding (1)
DriedRodentFaeces2	0	137	.000
	1	209	1.000
Farming	0	69	.000
	1	277	1.000
RatsInHouse	0	65	.000
	1	281	1.000
Male?	0	178	.000
	1	168	1.000

Block 0: Beginning Block

Classification Table[a,b]

Observed			Predicted: Renal_patient? 0	Predicted: Renal_patient? 1	Predicted: Percentage Correct
Step 0	Renal_patient?	0	242	0	100.0
		1	104	0	.0
	Overall Percentage				69.9

a. Constant is included in the model.

b. The cut value is .500

Variables in the Equation

		B	S.E.	Wald	df	Sig.	Exp(B)
Step 0	Constant	-.845	.117	51.882	1	.000	.430

Variables not in the Equation

			Score	df	Sig.
Step 0	Variables	Q2_Age	56.314	1	.000
	Overall Statistics		56.314	1	.000

Block 1: Method = Enter

Omnibus Tests of Model Coefficients

		Chi-square	df	Sig.
Step 1	Step	60.633	1	.000
	Block	60.633	1	.000
	Model	60.633	1	.000

Model Summary

Step	-2 Log likelihood	Cox & Snell R Square	Nagelkerke R Square
1	362.424[a]	.161	.228

a. Estimation terminated at iteration number 5 because parameter estimates changed by less than .001.

Hosmer and Lemeshow Test

Step	Chi-square	df	Sig.
1	10.651	8	.222

Contingency Table for Hosmer and Lemeshow Test

		Renal_patient? = 0		Renal_patient? = 1		
		Observed	Expected	Observed	Expected	Total
Step 1	1	35	32.908	0	2.092	35
	2	33	32.923	4	4.077	37
	3	32	31.761	5	5.239	37
	4	32	30.525	6	7.475	38
	5	24	26.981	12	9.019	36
	6	23	20.591	7	9.409	30
	7	18	21.441	16	12.559	34
	8	16	17.450	15	13.550	31
	9	15	17.951	22	19.049	37
	10	14	9.468	17	21.532	31

Classification Table[a]

			Predicted		
			Renal_patient?		Percentage Correct
	Observed		0	1	
Step 1	Renal_patient?	0	222	20	91.7
		1	75	29	27.9
	Overall Percentage				72.5

a. The cut value is .500

Variables in the Equation

		B	S.E.	Wald	df	Sig.	Exp(B)	95% C.I...
								Lower
Step 1[a]	Q2_Age	.070	.010	46.995	1	.000	1.072	1.051
	Constant	-4.481	.566	62.679	1	.000	.011	

Variables in the Equation

		95% C.I....
		Upper
Step 1[a]	Q2_Age	1.094
	Constant	

a. Variable(s) entered on step 1: Q2_Age.

Block 2: Method = Enter

Omnibus Tests of Model Coefficients

		Chi-square	df	Sig.
Step 1	Step	16.242	1	.000
	Block	16.242	1	.000
	Model	76.875	2	.000

Model Summary

Step	-2 Log likelihood	Cox & Snell R Square	Nagelkerke R Square
1	346.182[a]	.199	.282

a. Estimation terminated at iteration number 5 because parameter estimates changed by less than .001.

Hosmer and Lemeshow Test

Step	Chi-square	df	Sig.
1	16.315	8	.038

Contingency Table for Hosmer and Lemeshow Test

		Renal_patient? = 0		Renal_patient? = 1		
		Observed	Expected	Observed	Expected	Total
Step 1	1	35	33.492	0	1.508	35
	2	33	33.221	3	2.779	36
	3	31	30.402	4	4.598	35
	4	30	27.623	4	6.377	34
	5	29	27.494	7	8.506	36
	6	23	25.283	13	10.717	36
	7	21	23.350	15	12.650	36
	8	18	20.181	18	15.819	36
	9	10	15.098	26	20.902	36
	10	12	5.855	14	20.145	26

Classification Table[a]

Observed			Predicted: Renal_patient? 0	Predicted: Renal_patient? 1	Predicted: Percentage Correct
Step 1	Renal_patient?	0	220	22	90.9
		1	64	40	38.5
	Overall Percentage				75.1

a. The cut value is .500

Variables in the Equation

		B	S.E.	Wald	df	Sig.	Exp(B)	95% C.I.... Lower	95% C.I.... Upper
Step 1[a]	Q2_Age	.070	.011	43.091	1	.000	1.073	1.050	1.095
	Q3_Gender(1)	1.059	.269	15.519	1	.000	2.884	1.703	4.884
	Constant	-5.058	.622	66.219	1	.000	.006		

a. Variable(s) entered on step 1: Q3_Gender.

Block 3: Method = Enter

Omnibus Tests of Model Coefficients

		Chi-square	df	Sig.
Step 1	Step	6.437	1	.011
	Block	6.437	1	.011
	Model	83.312	3	.000

Model Summary

Step	-2 Log likelihood	Cox & Snell R Square	Nagelkerke R Square
1	339.744[a]	.214	.303

a. Estimation terminated at iteration number 5 because parameter estimates changed by less than .001.

Hosmer and Lemeshow Test

Step	Chi-square	df	Sig.
1	13.255	8	.103

Contingency Table for Hosmer and Lemeshow Test

		Renal_patient? = 0		Renal_patient? = 1		
		Observed	Expected	Observed	Expected	Total
Step 1	1	34	34.098	1	.902	35
	2	33	32.478	2	2.522	35
	3	33	31.118	2	3.882	35
	4	29	28.779	6	6.221	35
	5	30	26.618	5	8.382	35
	6	23	24.990	13	11.010	36
	7	22	23.280	15	13.720	37
	8	17	19.330	18	15.670	35
	9	10	15.411	27	21.589	37
	10	11	5.899	15	20.101	26

Classification Table[a]

			Predicted		
			Renal_patient?		Percentage Correct
	Observed		0	1	
Step 1	Renal_patient?	0	218	24	90.1
		1	62	42	40.4
	Overall Percentage				75.1

a. The cut value is .500

Variables in the Equation

		B	S.E.	Wald	df	Sig.	Exp(B)	95% C.I....
								Lower
Step 1[a]	Q2_Age	.067	.011	37.591	1	.000	1.069	1.046
	Q3_Gender(1)	.957	.273	12.294	1	.000	2.603	1.525
	Farming(1)	1.113	.478	5.410	1	.020	3.043	1.191
	Constant	-5.796	.741	61.160	1	.000	.003	

Variables in the Equation

		95% C.I....
		Upper
Step 1[a]	Q2_Age	1.092
	Q3_Gender(1)	4.445
	Farming(1)	7.773
	Constant	

a. Variable(s) entered on step 1: Farming.

Block 4: Method = Enter

Omnibus Tests of Model Coefficients

		Chi-square	df	Sig.
Step 1	Step	.463	1	.496
	Block	.463	1	.496
	Model	83.775	4	.000

Model Summary

Step	-2 Log likelihood	Cox & Snell R Square	Nagelkerke R Square
1	339.282[a]	.215	.305

a. Estimation terminated at iteration number 5 because parameter estimates changed by less than .001.

Hosmer and Lemeshow Test

Step	Chi-square	df	Sig.
1	9.106	8	.333

Contingency Table for Hosmer and Lemeshow Test

		Renal_patient? = 0		Renal_patient? = 1		
		Observed	Expected	Observed	Expected	Total
Step 1	1	34	34.120	1	.880	35
	2	33	32.494	2	2.506	35
	3	33	31.086	2	3.914	35
	4	29	28.776	6	6.224	35
	5	30	25.975	4	8.025	34
	6	22	23.677	12	10.323	34
	7	20	23.466	17	13.534	37
	8	17	19.744	18	15.256	35
	9	13	15.113	22	19.887	35
	10	11	7.549	20	23.451	31

Classification Table[a]

Observed			Predicted: Renal_patient? 0	1	Percentage Correct
Step 1	Renal_patient?	0	220	22	90.9
		1	62	42	40.4
	Overall Percentage				75.7

a. The cut value is .500

Variables in the Equation

		B	S.E.	Wald	df	Sig.	Exp(B)
Step 1[a]	Q2_Age	.067	.011	37.683	1	.000	1.069
	Q3_Gender(1)	.963	.273	12.406	1	.000	2.619
	Farming(1)	1.127	.479	5.534	1	.019	3.086
	RatsInHouse(1)	-.235	.344	.468	1	.494	.790
	Constant	-5.635	.774	53.030	1	.000	.004

Variables in the Equation

		95% C.I.for EXP(B) Lower	Upper
Step 1[a]	Q2_Age	1.047	1.092
	Q3_Gender(1)	1.533	4.476
	Farming(1)	1.207	7.892
	RatsInHouse(1)	.402	1.552
	Constant		

a. Variable(s) entered on step 1: RatsInHouse.

Block 5: Method = Enter

Omnibus Tests of Model Coefficients

		Chi-square	df	Sig.
Step 1	Step	8.960	1	.003
	Block	8.960	1	.003
	Model	92.735	5	.000

Model Summary

Step	-2 Log likelihood	Cox & Snell R Square	Nagelkerke R Square
1	330.322[a]	.235	.333

a. Estimation terminated at iteration number 6 because parameter estimates changed by less than .001.

Hosmer and Lemeshow Test

Step	Chi-square	df	Sig.
1	6.813	8	.557

Contingency Table for Hosmer and Lemeshow Test

		Renal_patient? = 0		Renal_patient? = 1		
		Observed	Expected	Observed	Expected	Total
Step 1	1	33	34.358	2	.642	35
	2	34	33.005	1	1.995	35
	3	34	31.615	1	3.385	35
	4	30	29.418	5	5.582	35
	5	25	25.363	8	7.637	33
	6	24	24.504	11	10.496	35
	7	23	23.491	15	14.509	38
	8	17	18.391	17	15.609	34
	9	12	13.883	22	20.117	34
	10	10	7.973	22	24.027	32

Classification Table[a]

			Predicted		
			Renal_patient?		Percentage Correct
Observed			0	1	
Step 1	Renal_patient?	0	218	24	90.1
		1	58	46	44.2
	Overall Percentage				76.3

a. The cut value is .500

Variables in the Equation

		B	S.E.	Wald	df	Sig.	Exp(B)
Step 1[a]	Q2_Age	.071	.011	40.283	1	.000	1.074
	Q3_Gender(1)	1.007	.278	13.075	1	.000	2.737
	Farming(1)	1.012	.486	4.340	1	.037	2.751
	RatsInHouse(1)	-1.058	.462	5.249	1	.022	.347
	DriedRodentFaeces2(1)	1.106	.389	8.102	1	.004	3.023
	Constant	-5.867	.798	54.038	1	.000	.003

Variables in the Equation

		95% C.I.for EXP(B)	
		Lower	Upper
Step 1[a]	Q2_Age	1.051	1.098
	Q3_Gender(1)	1.586	4.723
	Farming(1)	1.062	7.127
	RatsInHouse(1)	.140	.858
	DriedRodentFaeces2(1)	1.411	6.475
	Constant		

a. Variable(s) entered on step 1: DriedRodentFaeces2.

Block 6: Method = Enter

Omnibus Tests of Model Coefficients

		Chi-square	df	Sig.
Step 1	Step	15.846	1	.000
	Block	15.846	1	.000
	Model	108.581	6	.000

Model Summary

Step	-2 Log likelihood	Cox & Snell R Square	Nagelkerke R Square
1	314.476[a]	.269	.382

a. Estimation terminated at iteration number 6 because parameter estimates changed by less than .001.

Hosmer and Lemeshow Test

Step	Chi-square	df	Sig.
1	12.536	8	.129

Contingency Table for Hosmer and Lemeshow Test

		Renal_patient? = 0		Renal_patient? = 1		
		Observed	Expected	Observed	Expected	Total
Step 1	1	33	34.466	2	.534	35
	2	34	33.424	1	1.576	35
	3	34	32.041	1	2.959	35
	4	29	29.807	6	5.193	35
	5	32	27.529	3	7.471	35
	6	24	24.989	11	10.011	35
	7	21	23.012	15	12.988	36
	8	15	18.327	20	16.673	35
	9	12	12.819	23	22.181	35
	10	8	5.585	22	24.415	30

Classification Table[a]

Observed			Predicted		
			Renal_patient?		Percentage Correct
			0	1	
Step 1	Renal_patient?	0	219	23	90.5
		1	49	55	52.9
	Overall Percentage				79.2

a. The cut value is .500

Variables in the Equation

		B	S.E.	Wald	df	Sig.	Exp(B)
Step 1[a]	Q2_Age	.069	.012	34.810	1	.000	1.071
	Q3_Gender(1)	.756	.291	6.753	1	.009	2.131
	Farming(1)	.917	.490	3.502	1	.061	2.503
	RatsInHouse(1)	-1.209	.474	6.509	1	.011	.299
	DriedRodentFaeces2(1)	1.250	.403	9.634	1	.002	3.491
	Hanta_positive	1.189	.301	15.634	1	.000	3.284
	Constant	-5.863	.818	51.438	1	.000	.003

Variables in the Equation

		95% C.I.for EXP(B)	
		Lower	Upper
Step 1[a]	Q2_Age	1.047	1.096
	Q3_Gender(1)	1.204	3.769
	Farming(1)	.957	6.543
	RatsInHouse(1)	.118	.756
	DriedRodentFaeces2(1)	1.585	7.689
	Hanta_positive	1.822	5.922
	Constant		

a. Variable(s) entered on step 1: Hanta_positive.
